# Narcissistic and Antisocial Personality Traits Are Both Encoded in the Triple Network: Connectomics Evidence

**DOI:** 10.1111/psyp.70130

**Published:** 2025-08-11

**Authors:** Khanitin Jornkokgoud, Richard Bakiaj, Peera Wongupparaj, Remo Job, Alessandro Grecucci

**Affiliations:** ^1^ Department of Research and Applied Psychology, Faculty of Education Burapha University Chon Buri Thailand; ^2^ Department of Psychology and Cognitive Sciences (DiPSCo) University of Trento Rovereto Italy; ^3^ Faculty of Psychology Chulalongkorn University Bangkok Thailand; ^4^ Centre for Medical Sciences (CISMed) University of Trento Trento Italy

**Keywords:** default mode network, fronto‐parietal network, graph‐based theory, narcissism, salience network

## Abstract

The neural bases of narcissistic and antisocial traits remain under debate. A key question is whether these traits are encoded within the triple network—comprising the default mode (DMN), salience (SN), and fronto‐parietal (FPN) networks—and whether they impact these networks similarly. We conducted connectome‐based analyses on resting‐state fMRI data from 183 participants, examining graph‐theoretical metrics in the DMN, SN, and FPN, using the visual and sensorimotor networks as controls. Predictive models of narcissistic and antisocial traits were developed using stepwise multiple regression and Random Forest regression to ensure generalizability. Seed‐based analyses were conducted using regions identified by these models. Our findings revealed clear involvement of the triple network in both traits, supporting a shared neural substrate. Both traits were negatively predicted by the anterior cingulate cortex of the SN, reflecting reduced danger awareness and increased risky behaviors. Conversely, both were positively predicted by the lateral prefrontal cortex of the FPN, suggesting augmented strategic thinking to manipulate others and increased planning skills to achieve personal goals. Besides similarities, there were differences. Specific hubs of the DMN were positively associated with narcissism but negatively with antisocial traits, possibly explaining their differences in self‐reflection and thinking about the self, largely present in the former, but usually reduced in the latter. These results expand on prior evidence linking the triple network to personality traits and suggest both shared and distinct neural mechanisms for narcissism and antisociality. These findings may help inform the development of biomarkers for personality pathology and guide biologically informed interventions.

## Impact Statement

The neural underpinnings of narcissistic and antisocial traits through a graph‐based network analysis identified shared and distinct roles of the triple brain network in these personalities. By highlighting network‐specific contributions to self‐reflection, emotion regulation, and cognitive control, these findings advance our understanding of personality pathology and offer potential targets for clinical intervention.

## 1 Introduction

Narcissism exists on a continuum, ranging from subtle, sub‐clinical traits to the pathological condition of Narcissistic Personality Disorder (NPD), with each manifestation impacting mental health and interpersonal relationships to varying degrees (Nenadić et al. 2021). This continuum reflects alterations in psychological components such as self‐esteem regulation, self‐coherence, and empathy (Miller et al. 2011; Ronningstam 2011). At the extreme end of the continuum lies NPD, marked by dominance, inflated self‐importance, and anger—hallmarks of grandiose narcissism (Miller et al. 2011). In contrast, vulnerable narcissism is characterized by insecurity, hypersensitivity, and shame, often concealed through defensive behaviors (Miller et al. 2011). Narcissistic traits can also be found in the general population without meeting the criteria for a formal diagnosis. One intriguing, but poorly understood aspect is that narcissistic traits often combine with antisocial traits. Antisocial personality traits (APT) fall along a continuum of behaviors that violate social norms; at their extreme, they fulfill the DSM‐5 criteria for Antisocial Personality Disorder (ASPD). ASPD is diagnosed in individuals aged ≥ 18 years who showed conduct disorder symptoms before age 15 and who, since adolescence, display a persistent disregard for the rights of others. A diagnosis requires endorsement of at least three of seven behavioral indicators: (1) habitual illegal acts; (2) chronic deceit or manipulation; (3) impulsivity or poor future planning; (4) irritability and recurrent physical aggression; (5) reckless indifference to personal or others' safety; (6) persistent irresponsibility in work or financial matters; and (7) a lack of remorse for harm inflicted. These patterns must not occur exclusively during episodes of schizophrenia or bipolar disorder (American Psychiatric Association 2013).

Accurately classifying personality disorders remains a key challenge, especially given the overlap between traits such as narcissism and antisociality. Both the fifth edition, text revision, of the Diagnostic and Statistical Manual of Mental Disorders (DSM‐5‐TR) and the eleventh revision of the International Classification of Diseases (ICD‐11) provide frameworks for diagnosing personality disorders, traditionally using categorical models. However, due to high symptom overlap and limited clinical precision, dimensional models have been proposed and increasingly adopted, emphasizing traits on a continuum of severity (American Psychiatric Association 2013; Ashton 2013; Kotov et al. 2022; Tyrer et al. 2015). Narcissistic and antisocial personalities, although distinct in classification, often overlap. Shared features include dominance, inflated self‐importance, anger, manipulation, and impulsivity, and both traits frequently co‐occur in the same individuals. Previous research has indeed found that antisocial traits can predict narcissistic traits and vice versa (Jornkokgoud et al. 2023). Similarly, individuals with antisocial traits may be considered as exhibiting a more extreme form of narcissism (Kernberg 1992; Miller et al. 2017). The frequent co‐occurrence of narcissistic and antisocial traits has led some researchers to conceptualize them as part of broader patterns, such as the Dark Triad or malignant narcissism (Kernberg 1989, 1992; Paulhus and Williams 2002). These conceptual frameworks underscore the need to investigate shared neurobiological mechanisms. Understanding how these traits are organized at the neural level may offer crucial insights into the dimensional nature of personality pathology.

Recent neuroimaging studies, including our own, have begun to explore the neural underpinnings of personality traits through the lens of dimensional models, suggesting that brain‐based markers may enhance our understanding of personality psychopathology (DeYoung et al. 2022; Grecucci et al. 2022; Jauk and Kanske 2021; Jornkokgoud et al. 2024, 2023; Kotov et al. 2022; Langerbeck et al. 2023) by linking specific brain patterns to trait dimensions, potentially enabling early identification before traits reach clinical severity (Luo et al. 2022). Neuroscience applied to personality offered valuable insights into the underlying mechanisms of narcissism (Cao et al. 2022; DeYoung et al. 2022; Fan et al. 2011; Feng et al. 2018; Jauk et al. 2017) and antisociality (Blair 2010; Gregory et al. 2012; Jiang et al. 2016, 2013; Kumari et al. 2006, 2013, 2014; Tang et al. 2016). This neurobiological perspective supports a dimensional approach, highlighting the continuity of personality traits across both subclinical and clinical populations.

More recent studies have started clarifying the importance of functional connectivity, which yields more accurate behavioral predictions (Seguin et al. 2020). Resting‐state functional connectivity (RSFC) analyses have identified key nodes, including the amygdala, prefrontal cortex (PFC), and the anterior cingulate cortex (ACC), as crucial areas involved in the neural network of narcissistic traits (Feng et al. 2018). Complementing these findings, structural MRI studies have revealed significant variations in gray matter (GM) within the prefrontal and insular cortices, as well as alterations in white matter (WM) microstructure in individuals with narcissism or NPD (Jornkokgoud et al. 2024, 2023; Nenadic et al. 2015). Similarly, in individuals with ASPD, RSFC has revealed reduced activity in the posterior cerebellum and middle frontal gyrus, along with increased activity in the inferior temporal, middle occipital, and inferior occipital gyri (Kumari et al. 2006; Liu et al. 2014). Additionally, psychopathy and antisocial traits have been linked to widespread structural impairments across multiple brain regions. For instance, notable reductions in GM have been observed in several brain regions, including the frontopolar cortex, orbitofrontal cortex (OFC), frontal gyri, ACC, medial prefrontal cortex (MPFC), superior temporal gyrus, superior temporal sulcus, sensory–motor area, and rectal gyrus (Blair 2010; Gregory et al. 2012; Jiang et al. 2016, 2013; Kumari et al. 2006, 2013, 2014; Narayan et al. 2007; Raine et al. 2011; Tang et al. 2016). These findings highlight the complex neurobiological underpinnings of narcissistic and antisocial traits and their potential implications for understanding NPD and ASPD.

Given the frequent co‐occurrence of narcissistic and antisocial traits in certain individuals, which often leads to severe personality disturbances, it is crucial to investigate the common neural mechanisms underlying these traits. For instance, individuals with NPD exhibit altered RSFC patterns between brain regions in the default mode network (DMN) compared to healthy controls (Cao et al. 2022). This disrupted connectivity, especially between the DMN and cognitive networks, significantly predicts narcissistic traits (Cao et al. 2022; Jornkokgoud et al. 2024). Previous research has highlighted the interaction of key brain networks, particularly the triple network, which includes the salience network (SN), central executive network or fronto‐parietal network (FPN), and DMN, in other personality disorders included in the cluster B domain, such as borderline (Aguilar‐Ortiz et al. 2020; Amiri et al. 2023; Doll et al. 2013; Krause‐Utz et al. 2014; Tang et al. 2016). Nonetheless, previous studies focusing on macro networks in NPT or NPD have yielded limited results, as discussed in Jornkokgoud et al. (2024). Furthermore, collective evidence suggests that alterations in the DMN, SN, and FPN (the triple network) may contribute not only to narcissistic traits but also to antisocial traits.

Notably, most studies on the triple network and personality traits have relied on ROI‐to‐ROI or seed‐based connectivity to assess inter‐regional communication. However, the internal organization of these networks remains largely unexplored. Graph‐based approaches address this gap by modeling brain regions (nodes) and their connections (edges) (Faskowitz et al. 2022; Sporns 2018). Specifically, the graph‐based network method is a powerful tool for studying both functional and network connectivity, offering a detailed topological analysis of the brain's network functionality (Farahani et al. 2019; Sporns 2018). Key metrics include nodal global efficiency (how efficiently a brain region communicates with all others), local efficiency (integration within a region's immediate network), average path length (the typical number of functional steps between brain regions), betweenness centrality (how often a region connects otherwise distant areas), and eccentricity (how far a region is from the most remote part of the network, reflecting its centrality) (Farahani et al. 2019; Sporns 2018). Moreover, this approach can provide novel insights into neurobiological mechanisms underlying cognition, behavior, and brain disorders (Farahani et al. 2019). The graph‐based network analysis of RSFC data, particularly techniques derived from graph theory, has become widely used for analyzing brain network connectivity (Islam et al. 2018; Rubinov and Sporns 2010; Sporns 2015, 2018). With this method, many studies have found alterations in neurological disorders (Aracil‐Bolaños et al. 2022; Wolf et al. 2011; Xu et al. 2016). With regard to narcissistic personality, as far as we know, only one study has reported abnormal topology within the DMN in young male patients using this method (Cao et al. 2022). In addition, individuals with antisocial traits also exhibit altered RSFC topology, primarily in the DMN, with increased clustering and reduced betweenness centrality in several frontal, parietal, and temporal regions (Tang et al. 2016). However, these studies were limited to a small sample size (i.e., ranging from 19 to 64) and focused solely on the DMN. Therefore, a more balanced exploration of multiple personality traits through graph‐based network analysis could provide deeper insights into the shared and distinct neural mechanisms of these traits.

To address these gaps in the literature, the main objective of the current investigation is to identify and characterize the functionality of macro‐networks in both narcissistic and antisocial personality. We hypothesize that abnormalities within the DMN, SN, and FPN will be predictive of both traits. Specifically, we expected regional topological measures (such as the betweenness and eccentricity), as well as global topological measures (such as nodal global efficiency, local efficiency, and average path length) within the DMN, SN, and FPN, to predict both narcissistic and antisocial personality traits by using multiple regression with a stepwise method. In addition, a supervised machine learning approach known as random forest will be used to build predictive models of narcissistic and antisocial traits based on graph theoretical metrics. Random forest modeling builds an ensemble of decision trees predicting the result of interest for regression problems by using random subsets of features (Breiman 2001; Breiman et al. 2017). It is well‐suited for capturing non‐linear relationships and evaluating variable importance while being robust to multicollinearity and overfitting (Breiman 2001; Breiman et al. 2017; Matsuki et al. 2016). This method will enable us to test the generalizability of our results to new, previously unobserved cases (Grecucci et al. 2023).

Concerning the triple network, previous theoretical models have implicated large‐scale brain networks in the expression of psychopathy (Hamilton et al. 2015). More recently, the Impaired Integration (II) model proposed by Hamilton et al. (2015) suggests that psychopathy involves reduced coordination and impaired switching between large‐scale networks, particularly the SN, DMN, and FPN, leading to deficits in socioemotional processing; supporting this, scholars found that individuals with high psychopathic traits exhibit impaired switching between the DMN and FPN, pointing to dysfunction in the SN's role as a network switcher (Deming et al. 2023; Kng et al. 2025). More specifically, Blair's Integrated Emotion Systems model highlights dysfunction in the amygdala and orbitofrontal cortex, key nodes of the SN, as contributing to deficits in empathy and moral reasoning (Blair 2003, 2007). Moreover, Kiehl's paralimbic dysfunction hypothesis extends this view, implicating a broader network involving the ACC, MPFC, and temporal regions, many of which overlap with the DMN, in psychopathy (Kiehl 2006; Langerbeck et al. 2023). Furthermore, the FPN, crucial for executive functions like impulse control and decision‐making, has been implicated in psychopathy due to its role in regulating antisocial impulses and goal‐directed behavior; dysfunction in this network may underlie core features of psychopathy such as impulsivity and poor behavioral regulation (Hamilton et al. 2015; Kng et al. 2025). These models provide a compelling rationale for investigating functional and topological alterations within the DMN, SN, and FPN in individuals with narcissistic and antisocial traits.

However, we also expect some differences in local and global metrics in the two personalities. Narcissistic individuals spend a lot of time reflecting on themselves to compensate for self‐esteem with fantasies of power and importance (Di Pierro et al. 2016) whereas antisocial individuals are less reflective and more oriented to engage in antisocial behavior and violate the rights of others (De Wit‐De Visser et al. 2023). Indeed, antisocial individuals are typically impulsive and do not reflect on themselves or the consequences of their actions (De Wit‐De Visser et al. 2023; Korponay et al. 2017). This may be reflected in differences in how the DMN is related to these disorders. We predict that higher narcissistic traits will be associated with increased functionality, whereas antisocial traits will show a decreased DMN functionality.

To further understand the communications between the main regions of the macro‐networks and other regions of the brain, seed‐based connectivity will be considered too. The aim of seed‐based analysis was to understand how key hubs within the triple network identified in graph theory analyses may influence other brain regions. By combining these two analyses, we hope to enhance our understanding of local and distributed abnormal brain functionality in individuals with narcissistic and antisocial traits. We expect to find results that are coherent with the ones detected with the topological analysis.

## 2 Materials and Methods

### 2.1 Participants

We utilized data from the MPI‐Leipzig Mind Brain–Body dataset, which is accessible through the OpenNeuro database (Accession Number: ds000221) (Babayan et al. 2020). This dataset encompasses MRI and behavioral data gathered from 318 participants who participated in the project conducted by the Max Planck Institute (MPI) of Human Cognitive and Brain Sciences in Leipzig. The study was carried out under the authorization of the ethics committee at the University of Leipzig (Protocol ID: 154/13‐ff) (Babayan et al. 2019).

For the purposes of this study, we rigorously selected data from individuals who participated in the LEMON and Neuroanatomy & Connectivity Protocols according to specific selection criteria. These criteria encompassed medical eligibility for magnetic resonance sessions, availability of structural T1‐weighted images and functional MRI, and completion of the Personality Styles and Disorders Inventory (PSDI), with a focus on the narcissistic and antisocial scales. Participants aged 70 years or older were excluded to minimize potential confounding effects of age‐related alterations in brain functional connectivity or topography (Li et al. 2021; Qin and Basak 2020; Veréb et al. 2023).

The final sample comprised 183 healthy participants, including 89 females and 94 males. PSDI scores for narcissistic traits (*t* = 0.89, *p* = 0.37) and antisocial traits (*t* = 0.89, *p* = 0.39) did not differ significantly between genders. The participants' ages ranged from 22 to 68 years, with a mean age of 32.58 years (SD = 14.04 years). Age distribution was also balanced between genders (*t* = −0.82, *p* = 0.41).

### 2.2 Personality Styles and Disorders Inventory (PSDI)

To assess personality traits, we used the PSDI (German version: Persönlichkeits‐Stil‐und Störungs‐Inventar, PSSI), a validated self‐report inventory that measures various personality styles and can provide insights into potential personality disorders, particularly when extreme scores are observed. Developed and revised by Kuhl and Kazén (2009), the inventory consists of 140 items organized into 14 subscales. In our study, we focused specifically on the narcissistic (Mean score = 12.14, SD = ±4.76) and antisocial (Mean score = 12.92, SD = ±4.40) subscales. The scores of the two subscales are correlated (*r* = 0.44, *p* < 0.01).

The PSDI shows a robust network of theoretically coherent relationships with a large number of clinical and non‐clinical behavioral characteristics (e.g., suicidality, depression, psychosomatic symptoms, the Big Five personality traits, and the sixteen personality factors). This extensive network of associations supports the construct validity of the inventory. Furthermore, the PSDI employs objective scoring procedures and statistical analyses, and its consistency coefficients (Cronbach's alpha) range between α = 0.73 and 0.85. Test–retest coefficients vary between *r* = 0.68 and 0.83, indicating good test–retest reliability. The construct validity of the inventory is considered acceptable for both clinical and non‐clinical behaviors (Kuhl and Kazén 2009).

### 2.3 MRI Data Acquisition

The MPI‐Leipzig Mind Brain–Body dataset comprises quantitative T1‐weighted, functional, resting state, and diffusion‐weighted images acquired at the Day Clinic for Cognitive Neurology of the University Clinic Leipzig and the Max Planck Institute for Human and Cognitive and Brain Sciences (MPI CBS) in Leipzig, Germany (Babayan et al. 2019). For the purpose of our research, we only considered the T1‐weighted images. Magnetic Resonance Imaging (MRI) was performed on a 3 T Siemens MAGNETOM Verio scanner (Siemens Healthcare GmbH, Erlangen, Germany) with a 32‐channel head coil. The MP2RAGE sequence consisted of the following parameters: sagittal acquisition orientation, one 3D volume with 176 slices, TR = 5000 ms, TE = 2.92 ms, TI1 = 700 ms, TI2 = 2500 ms, FA1 = 4°, FA2 = 5°, pre‐scan normalization, echo spacing = 6.9 ms, bandwidth = 240 Hz/pixel, FOV = 256 mm, voxel size = 1 mm isotropic, GRAPPA acceleration factor = 3, slice order = interleaved, duration = 8 min 22 s.

### 2.4 fMRI Data Pre‐Processing

Data pre‐processing was performed using CONN (version 22) (Nieto‐Castanon and Whitfield‐Gabrieli 2022; Whitfield‐Gabrieli and Nieto‐Castanon 2012), SPM 12, and the MATLAB Toolbox (version 2021b). First, CONN's default pre‐processing pipeline uses SPM12's default parameters. This pipeline encompassed several stages: functional realignment and unwarping, translation and centering, conservative functional outlier detection, direct segmentation and normalization of functional data (1 mm resolution), translation and centering of structural data, segmentation and normalization of structural data (2.4 mm resolution), and lastly, spatial smoothing of functional and structural data using an 8 mm Gaussian kernel. Subsequently, the denoising phase was conducted. The aim of this phase is to pinpoint and eliminate confounding variables and artifacts from the estimated BOLD signal. These factors arise from three distinct sources: the BOLD signal originating from masks of white matter or cerebrospinal fluid, parameters and outliers defined during the pre‐processing step, and an estimation of the subjects' motion parameters.

In addition, functional data were denoised using a standard denoising pipeline, including the regression of potential confounding effects characterized by white matter timeseries, cerebrospinal fluid (CSF) timeseries motion parameters and their first‐order derivatives, outlier scans (below 79 factors), session effects and their first‐order derivatives, and linear trends within each functional run, followed by bandpass frequency filtering of the BOLD timeseries between 0.008 Hz and 0.09 Hz. CompCor noise components within white matter and CSF were estimated by computing the average BOLD signal as well as the largest principal components orthogonal to the BOLD average, motion parameters, and outlier scans within each subject's eroded segmentation masks. From the number of noise terms included in this denoising strategy, the effective degrees of freedom of the BOLD signal after denoising were estimated to range from 181.1 to 207 (average 205.3) across all subjects. We assessed motion quality by examining individual mean framewise displacement (FD) values. Seven participants exceeded the motion threshold for mean FD (ranging from 0.28 to 0.39 mm), based on the outlier detection method used in CONN's Artifact Detection Toolbox. However, none exceeded the commonly used exclusion threshold of 0.5 mm for mean FD (Power et al. 2014).

### 2.5 Connectivity Analysis

At the first‐level analysis, ROI‐to‐ROI connectivity (RRC) matrices were performed using the CONN toolbox (Whitfield‐Gabrieli and Nieto‐Castanon 2012). A total of 164 regions of interest (ROIs) were used for the analysis. This included 132 ROIs derived from the Harvard‐Oxford atlases implemented in the FMRIB Software Library (FSL), comprising 91 cortical and 15 subcortical regions, along with 26 cerebellar ROIs from the Automated Anatomical Labeling (AAL) atlas (Desikan et al. 2006). An additional 32 ROIs were functionally defined using ICA‐based network parcellations provided by the CONN toolbox, representing canonical brain networks such as the default mode, salience, sensorimotor, dorsal attention, frontoparietal, language, visual, and cerebellar networks (Nieto‐Castanon and Whitfield‐Gabrieli 2022). This combined approach has been used in previous studies to integrate anatomical coverage and functional specificity (Maleki et al. 2022; Yorita et al. 2024). Moreover, functional connectivity strength was computed as Fisher‐transformed bivariate correlation coefficients from a weighted general linear model (GLM) (Nieto‐Castanon 2020), capturing the association between the BOLD time‐series of each pair of network nodes.

### 2.6 Graph Theory Analysis

The CONN toolbox was utilized to analyze graph theoretical metrics at the second level (Whitfield‐Gabrieli and Nieto‐Castanon 2012). Our analyses incorporate nodes from 164 ROIs (Barillaro et al. 2024; Yorita et al. 2024). In addition, the threshold for the ROI‐to‐ROI connectivity matrix for each subject is at a set level (cost = 0.15) (Park et al. 2023). We extracted only the five networks, including DMN, SN, FPN, visual, and sensorimotor, when deriving the graph‐theoretical metrics used in our predictive modeling. Graph theory measurements depend on hypothesis levels, such as global measures, subnetwork levels measured by module, or region levels measured by node. The theory integrates the concept of functional specialization with that of distributed processing, thereby enhancing our understanding of brain function. By mapping brain connectivity, this approach reveals unique connectivity fingerprints for different regions, predicting their functional roles and highlighting the organization of functional groupings within the brain (Rubinov and Sporns 2010; Sporns 2018). In this study, we focused on regional measurements commonly used to assess nodal centrality in human brain functional connectivity, such as betweenness, closeness, eigenvector, and eccentricity. These metrics quantify different aspects of a region's topological role, such as its influence over communication pathways (betweenness), accessibility within the network (closeness), connection to other influential regions (eigenvector), and the longest shortest path to any other node (eccentricity) (Boccaletti et al. 2006; Rubinov and Sporns 2010; Zuo et al. 2011). Additionally, we examined global topology metrics at the nodal level, including nodal global efficiency, local efficiency, average path length, and clustering coefficient. These metrics characterize how efficiently a given ROI exchanges information with all other regions in the brain network (nodal global efficiency and path length) or how well it is integrated within its immediate local neighborhood (local efficiency) (Boccaletti et al. 2006; Rubinov and Sporns 2010; Zuo et al. 2011).

### 2.7 Predictive Models

To explore predictors of narcissistic and antisocial trait scores, we employed two complementary regression approaches. Firstly, the study was conducted using multiple regression with a stepwise method in IBM SPSS version 25. In particular, predictors were included in global and regional metrics resulting from graph network analysis, including the DMN, the SN, and the FPN, as well as the visual and sensorimotor networks as control networks. For each predictor, we report the unstandardized regression coefficient (B), its standard error (SE), the 95% confidence interval (CI), standardized coefficient (β), t‐statistic, and *p*‐value. The B value indicates the expected change in the trait score per unit change in the predictor, while SE represents the precision of this estimate. Standardized coefficients (β) are also provided to allow for comparison across variables with different scales. Secondly, we conducted an additional analysis using Random Forest regression in Jeffreys's Amazing Statistics Program (JASP) version 0.19.3 to assess the robustness and predictive performance of the associations observed. We employed the “hold‐out” method to partition the sample into three subsets: training (70%), validation (15%), and test (15%). The model was optimized with respect to the out‐of‐bag mean squared error (MSE). The model underwent a structured process, first being trained, then validated, and tested. Feature importance was estimated using 5000 permutations to assess the contribution of each predictor variable while controlling for random fluctuations. Predictive performance was evaluated on the independent test set using root mean squared error (RMSE), mean absolute error (MAE), and coefficient of determination (*R* ^2^). In addition, plotting a three‐dimensional (3D) brain picture utilizing graph network results that identified ROIs or nodes that can predict narcissistic and antisocial behaviors was done using the CONN toolbox. Two‐sided correlations and uncorrected *p* < 0.05 were used to threshold the 3D‐rendered brain view of the analyzed network of functional connectivity.

### 2.8 Seed‐Based Analysis

Seed‐based functional connectivity analysis was performed using the CONN toolbox. We examined the between‐subject effects of narcissistic and antisocial scores to establish connections between whole ROIs and constructed network matrices that resulted from the previous step. Our seed‐to‐ROI connectivity analyses were not conducted in a fully exploratory or whole‐brain fashion. Rather, they were hypothesis‐driven and limited to a small number of seeds. The individual ROI maps were generated to include threshold ROI‐to‐ROI connections based on intensity, applying a two‐sided threshold for negative and positive seed levels. The significance of seed ROIs was determined through uncorrected *p* < 0.05. This strategy aligns with previous neuroimaging work in personality neuroscience, where connectivity analyses build on prior nodal findings (Cao et al. 2022; Soleimani et al. 2022). Finally, the survival ROIs of each seed region were visualized in 3D brain plots using the CONN toolbox. The 3D‐rendered view figures show the supra‐threshold ROI‐level findings that the connectivity contrast effect sizes (between the seed and each target) and uncorrected *p*‐values for the designated second‐level analysis were shown for each target ROI. These connectivity directions and intensity were demonstrated by the negative color in blue and the positive color in red (Nieto‐Castanon 2020) Figures 1 and 2.

**FIGURE 1:**
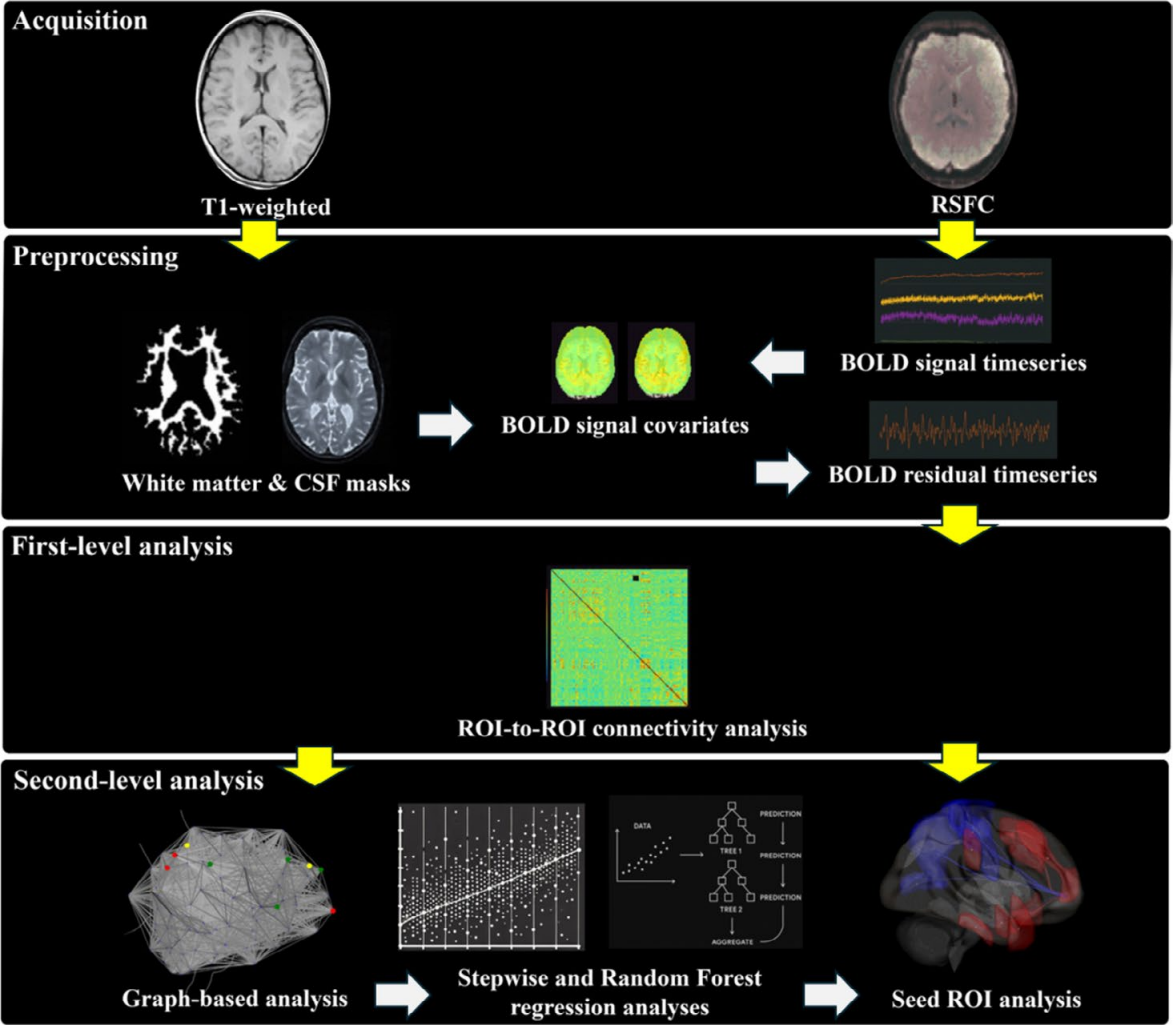
Data processing and analysis pipeline. Lines with an arrow indicate that the output of the previous step was supplied into the next step. The structural MRI (T1‐weighted) and resting‐state functional connectivity (RSFC) were acquired in the current study. The CONN toolbox was utilized to preprocess for both T1‐weighted and RSFC. T1‐weighted images were segmented into white matter and cerebrospinal fluid (CSF) masks. In the spatial domain, the blood‐oxygenation‐level‐dependent (BOLD) signal time series was computed from the results of slice‐timing, realignment, normalization, and smoothing. The BOLD residual timeseries were preprocessed at the temporal domain using the BOLD signal covariates, including segmented white matter and CSF masks, as well as BOLD signal timeseries. Analyses at the first‐level were performed using the ROI‐to‐ROI connectivity approach. The results of this step were then used for subsequent analyses, including graph theory, regression, and seed regions of interest (ROIs). Specific regions identified as survival nodes from the graph‐based analysis were included in the regression models, along with the seed ROIs.

**FIGURE 2:**
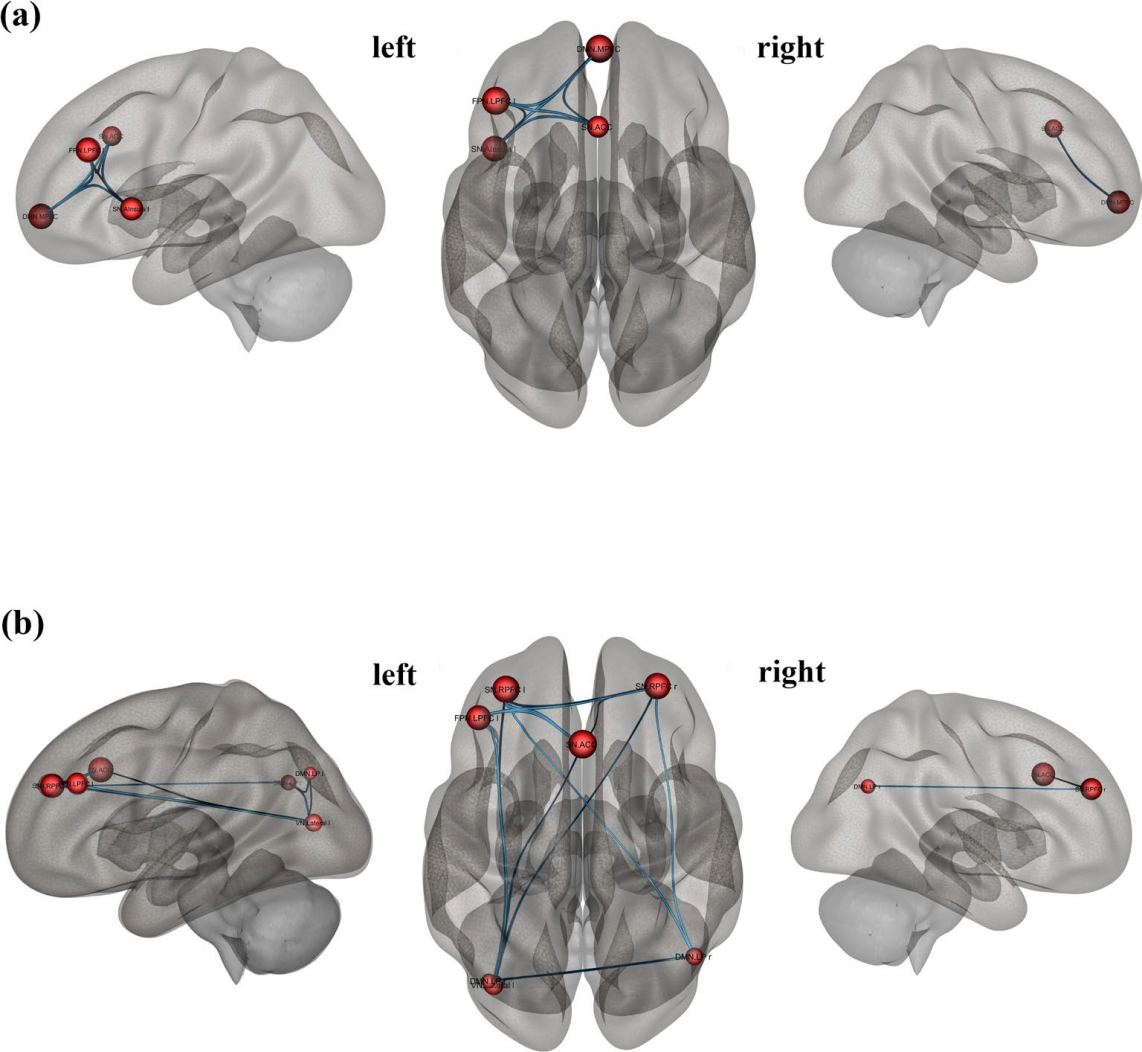
The brain network from graph‐based analysis, providing a 3D representation of the brain's connectivity concerning narcissistic traits (a) and antisocial traits (b). Seed ROIs are presented as nodes in red while two‐side correlations between nodes are presented in blue (*p*_unc_ < 0.05).

## 3 Results

### 3.1 Graph‐Based Network and Predictive Results

#### 3.1.1 Narcissistic Traits

The regression results indicated that the regional topological model accounted for a significant proportion of variance in narcissistic traits, *R* ^2^ = 0.11, (4, 95) = 6.96, 𝑝 < 0.001 *F* (4, 95) = 6.96, *p* < 0.001. Specifically, the eigenvector centrality of the MPFC within the DMN was a significant positive predictor (𝛽 = 0.17, 𝑡 = 2.29, 𝑝 = 0.02), while the eccentricity of the ACC region in the SN was a significant negative predictor (𝛽 = −0.17, 𝑡 = −2.35, 𝑝 = 0.02). Moreover, the betweenness centrality of the MPFC in the DMN (𝛽 = 0.15, 𝑡 = 2.06, 𝑝 = 0.04) and the betweenness centrality of the left anterior insula in the SN (𝛽 = −0.15, 𝑡= −2.06, 𝑝 = 0.04) were also significant predictors (See Table 1). Additionally, the Random Forest regression analysis supported the robustness of the findings. The algorithm indicated that the model explained approximately 13% of the variance in the narcissistic trait scores (MSE = 1.24; RMSE = 4.83; MAE = 3.83).

**TABLE 1:** Multiple regression analysis in regional topological matrices predicting narcissistic and antisocial traits.

Predictor	*B*	SE	B 95.0% CI [LL, UL]	*b*	*t*	*p*
*Narcissistic traits*						
Intercept	15.37	2.21	[11.01, 19.73]		6.96	< 0.001
DMN.MPFC, Eigenvector Centrality	1.49	0.65	[0.20, 2.77]	0.17	2.29	0.02
SN.ACC, Eccentricity	−1.22	0.52	[−2.24, −0.20]	−0.17	−2.35	0.02
DMN.MPFC, Betweenness Centrality	123.82	60.01	[5.39, 242.25]	0.15	2.06	0.04
SN.Ainsula l, Betweenness Centrality	−185.94	90.39	[−364.31, −7.58]	−0.15	−2.06	0.04
*Antisocial traits*						
Intercept	7.78	2.19	[3.46, 12.11]		3.55	< 0.001
SN.RPFC r, Betweenness Centrality	145.80	57.25	[32.84, 258.76]	0.18	2.55	0.01
SN.ACC Eccentricity	−1.71	0.50	[−2.69, −0.74]	−0.27	−3.46	< 0.01
VN.Lateral r, Eccentricity	1.28	0.50	[0.30, 2.27]	0.20	2.56	0.01

Furthermore, the overall global topological model was found to be significant, *R* ^2^ = 0.05, (2, 97) = 4.87, 𝑝 < 0.01. Although the intercept was not significant (𝑡 = −1.272, 𝑝 = 0.21), the nodal global efficiency of the MPFC in the DMN significantly predicted narcissistic traits (𝛽 = 0.16, 𝑡 = 2.15, 𝑝 = 0.03), as did the local efficiency of the left LPFC in the FPN (𝛽 = 0.15, 𝑡 = 2.08, 𝑝 = 0.04) (See Table 2). In addition, the Random Forest regression analysis revealed that the model accounted for approximately 8% of the variance in narcissistic trait scores (MSE = 1.37; RMSE = 5.01; MAE = 4.21).

**TABLE 2:** Multiple regression analysis in global topological matrices predicting narcissistic and antisocial traits.

Predictor	*B*	SE	B 95.0% CI [LL, UL]	*b*	*t*	*p*
*Narcissistic traits*						
Intercept	−8.025	6.31	[−20.475, 4.425]		−1.27	0.21
DMN.MPFC, Nodal Global Efficiency	17.20	7.99	[1.42, 32.97]	0.16	2.15	0.03
FPN.LPFC l, Local Efficiency	14.85	7.14	[0.76, 28.94]	0.15	2.08	0.04
*Antisocial traits*						
Intercept	8.41	5.09	[−1.64, 18.46]		1.65	0.10
DMN.LP r, Average Path Length	−1.93	0.75	[−3.41, −0.44]	−0.2	−2.56	0.01
DMN.LP l, Local Efficiency	−2.07	0.87	[−3.8, −0.35]	−0.19	−2.38	0.02
FPN.LPFC l, Average Path Length	5.50	2.09	[1.37, 9.62]	0.2	2.63	0.01
SN.RPFC l, Average Path Length	−3.12	1.49	[−6.06, −0.19]	−0.16	−2.10	0.04

#### 3.1.2 Antisocial Traits

The results of the regression indicated that the regional topological model explained a significant proportion of the variance in antisocial traits, *R* ^2^ = 0.11; *F* (3, 96) = 4.92; *p* < 0.001. The betweenness centrality of the right RPFC within the SN was found to be a significant positive predictor (𝛽 = 0.18, 𝑡 = 2.55, 𝑝 = 0.01), while the eccentricity of the ACC in the SN was found to be a significant negative predictor (𝛽 = −0.27, 𝑡 = −3.46, 𝑝 < 0.01). Moreover, the eccentricity of the right lateral part in the VN was also a significant positive predictor (𝛽 = 0.20, 𝑡 = 2.56, 𝑝 = 0.01), see Table 1. Additionally, the Random Forest regression analysis revealed that the model accounted for approximately 20% of the variance in antisocial trait scores (MSE = 1.06; RMSE = 3.83; MAE = 3.19).

Likewise, the global topological model was significant, *R* ^2^ = 0.10, (4, 95) = 5.73, 𝑝 < 0.001. Although the intercept was not significant (𝑡 = 1.65, 𝑝 = 0.10), the average path length of the right LP in the DMN (𝛽 = −0.20, 𝑡 = −2.56, 𝑝 = 0.01) and the local efficiency of the left LP in the DMN (𝛽 = −0.19, 𝑡 = −2.38, 𝑝 = 0.02) were significant negative predictors. Conversely, the average path length of the left LPFC in the FPN (𝛽 = 0.20, 𝑡 = 2.63, 𝑝 = 0.01) and the average path length of the left RPFC in the SN (𝛽 = −0.16, 𝑡 = −2.10, 𝑝 = 0.04) were significant predictors; see Table 2. Additionally, the Random Forest regression analysis revealed that the model accounted for approximately 9% of the variance in antisocial trait scores (MSE = 1.34; RMSE = 4.04; MAE = 3.32).

### 3.2 Seed ROI Results

When analyzing network metrics to predict narcissistic and antisocial traits, four key nodes were identified as significant predictors: DMN.MPFC, SN.ACC, SN.Ainsula l, and FPN.LPFC l. The following sections present the seed ROI results, which demonstrate that ROI targets were related to these seed sources/nodes, with a significant between‐subject effect on narcissistic and antisocial scores.

#### 3.2.1 Narcissistic Traits

**FIGURE 3:**
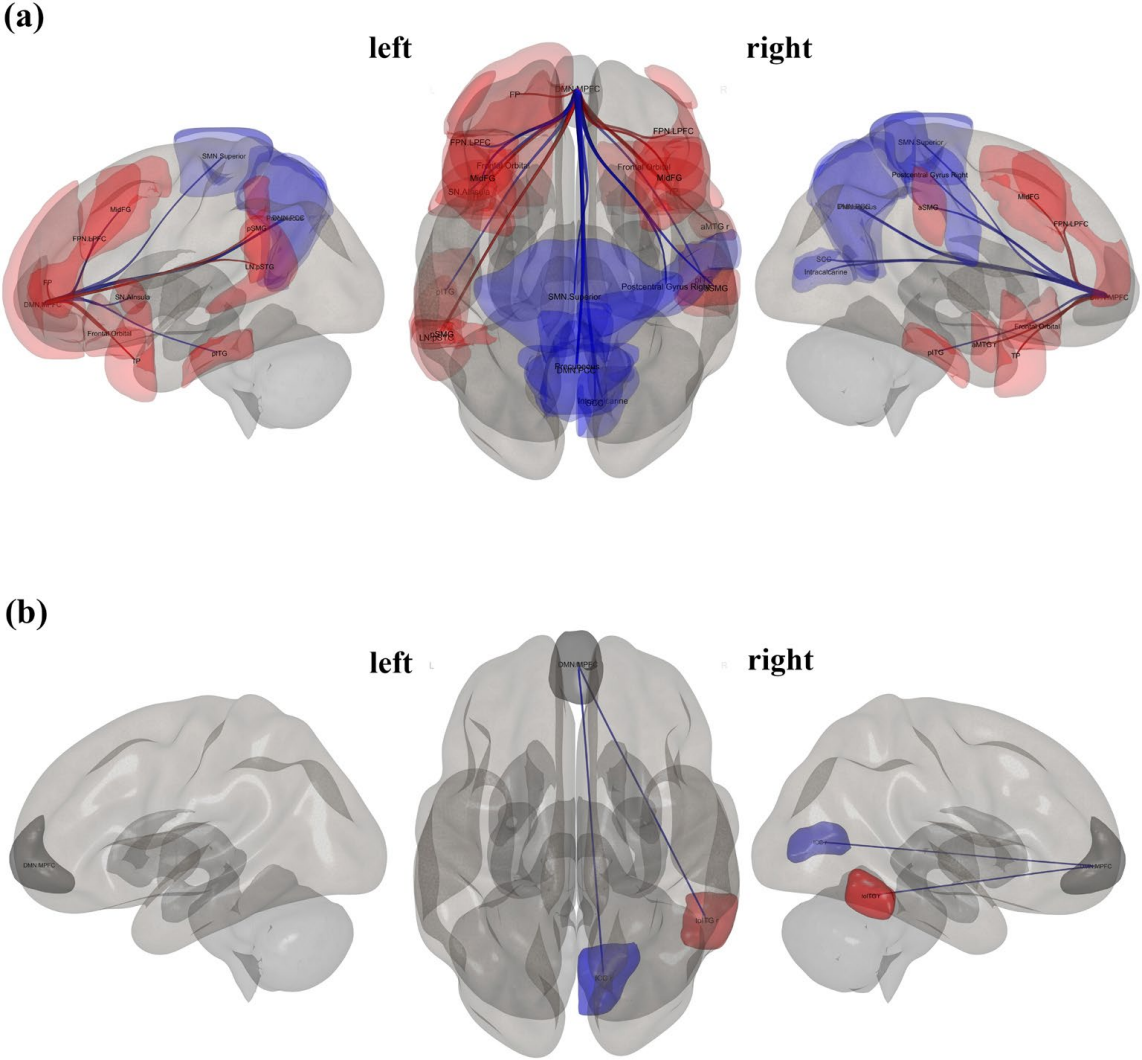
3D brain plots, specifically the seed ROI from DMN.MPFC, which is known to correlate with other regions (*p*_unc_ < 0.05). The positive color in red and negative color in blue indicate the strength and direction of these correlations, providing a visual representation of the brain's activity in relation to narcissistic traits (a) and antisocial traits (b).

**FIGURE 4:**
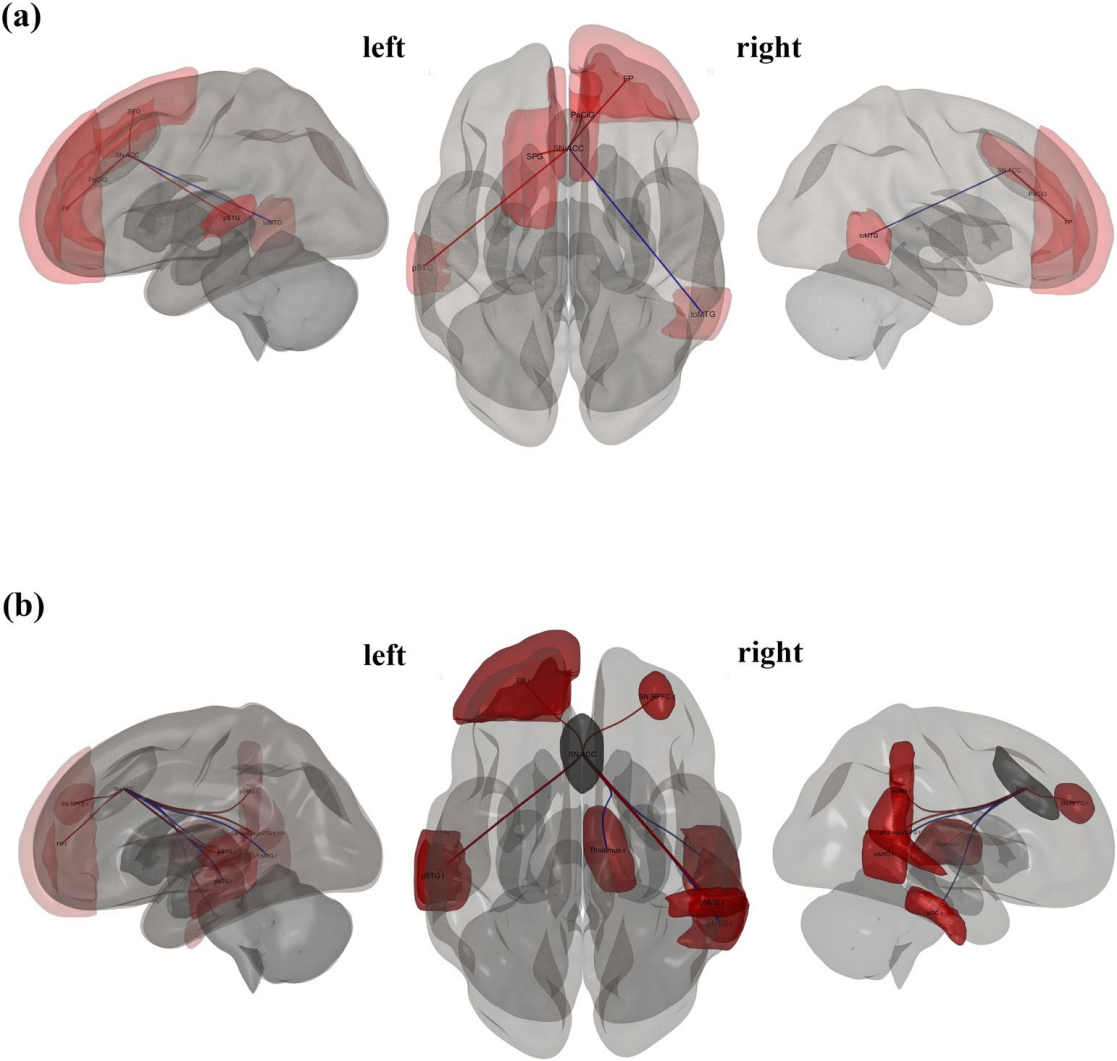
3D brain plots, specifically the seed ROI from SN.ACC, which is known to correlate with other regions (*p*_unc_ < 0.05). The positive correlation in red lines and negative correlation in blue lines indicate the strength and direction of these correlations, providing a visual representation of the brain's activity in relation to narcissistic traits (a) and antisocial traits (b).

**FIGURE 5:**
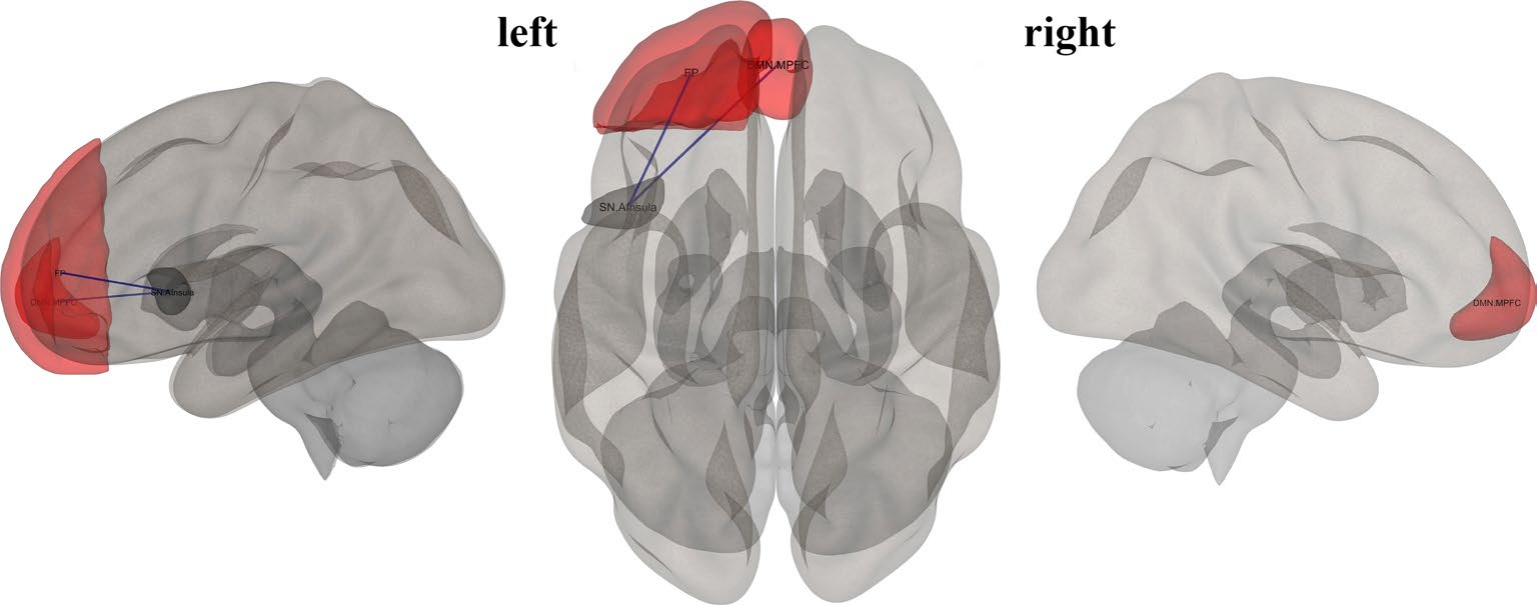
3D brain plots, specifically the seed ROI from SN.AInsula left, which is known to correlate with other regions (*p*_unc_ < 0.05). The positive correlation in red lines and negative correlation in blue lines indicate the strength and direction of these correlations, providing a visual representation of the brain's activity in relation to narcissistic traits.

**FIGURE 6:**
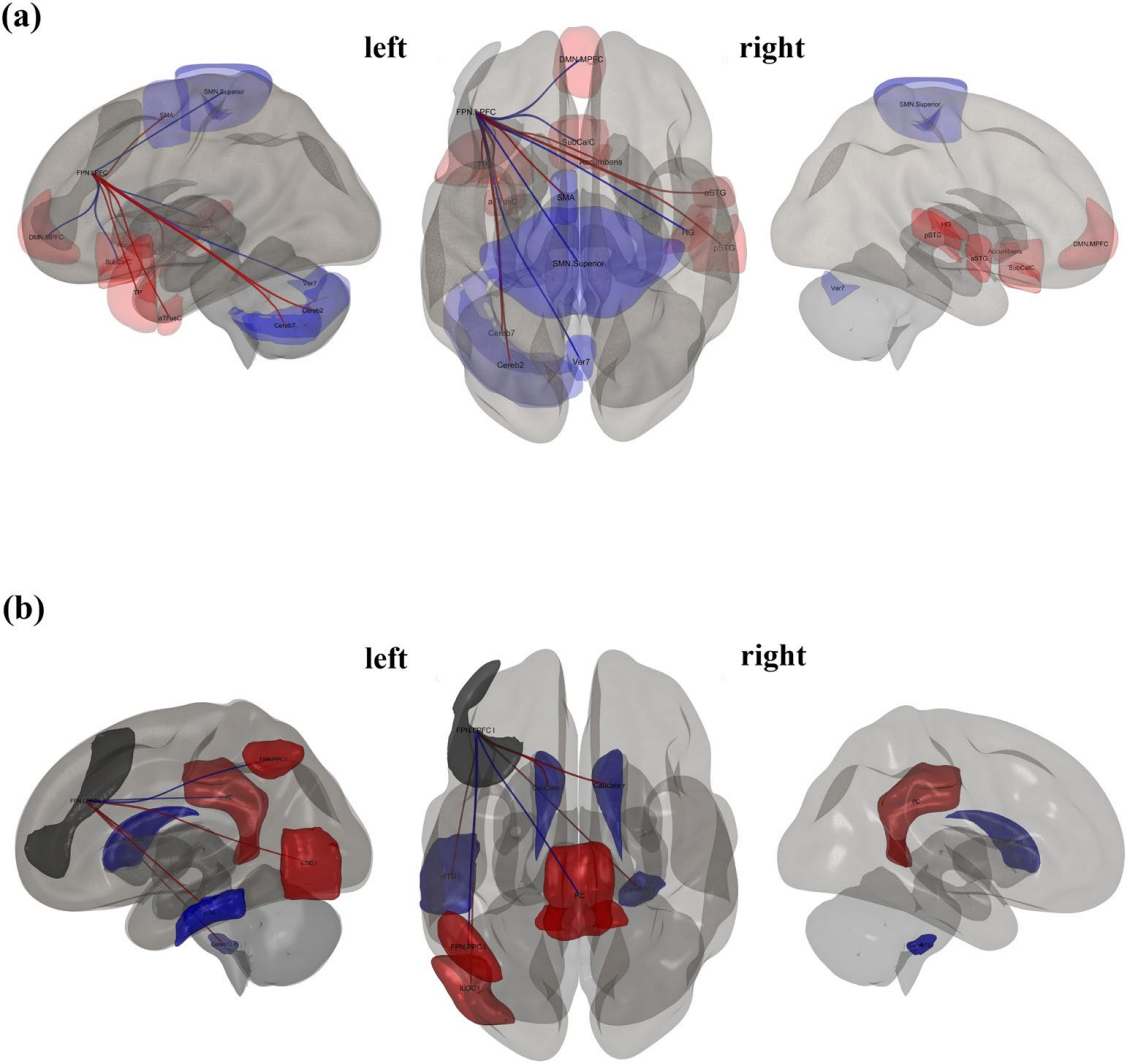
3D brain plots, specifically the seed ROI from FPN.LPFC, which is known to correlate with other regions (*p*_unc_ < 0.05). The positive correlation in red lines and negative correlation in blue lines indicate the strength and direction of these correlations, providing a visual representation of the brain's activity concerning narcissistic traits (a) and antisocial traits (b).

The seed regions and targets included ROIs that showed significant connections for narcissistic scores at an uncorrected *p*‐value of 0.05; see Table 3. Firstly, the MPFC of the DMN showed significant positive associations with multiple brain regions, including the left temporal pole, right frontal orbital cortex, and posterior inferior temporal gyrus on the left side (all *p*_unc_ < 0.01). Other positively associated areas (*p*_unc_ ranging from 0.02 to 0.04) included regions such as the right and left lateral prefrontal cortex, left supramarginal gyrus, and right middle frontal gyrus. Conversely, significant negative associations were found with regions such as the superior SMN, right supracalcarine cortex, and precuneus cortex (all *p*_unc_ = 0.01 or 0.02), as visualized in Figure 3a. Secondly, the ACC in the SN revealed significant positive associations with regions such as the right paracingulate gyrus and the right temporooccipital middle temporal gyrus (*p*‐unc < 0.01). Other regions, such as the posterior superior temporal gyrus on the left side and the right frontal pole, also showed positive associations (*p*_unc_ around 0.02). Additionally, the left superior frontal gyrus exhibited a significant positive association (*p*_unc_ = 0.04), as shown in Figure 4a. Thirdly, the Ainsula in the SN demonstrated significant positive associations with the left frontal lobe and the DMN medial prefrontal cortex (*p*_unc_ = 0.01 and 0.02, respectively), highlighting its specific connectivity and interaction with the DMN and other prefrontal regions, as visualized in Figure 5. Lastly, the LPFC in the FPN showed significant positive associations with several regions, including the right anterior superior temporal gyrus and the left anterior temporal fusiform cortex (*p*_unc_ < 0.01). Additional positive associations were found with areas such as the DMN medial prefrontal cortex, left temporal pole, and right Heschl's gyrus (*p*_unc_ ranging from 0.02 to 0.04). On the negative side, significant associations were observed with regions like the left juxtapositional lobule cortex and left cerebellum Crus2 (*p*_unc_ < 0.01), as well as other areas like the superior SMN and Vermis 7 (*p*_unc_ = 0.02 to 0.04), as visualized in Figure 6a.

#### 3.2.2 Antisocial Traits

**FIGURE 7:**
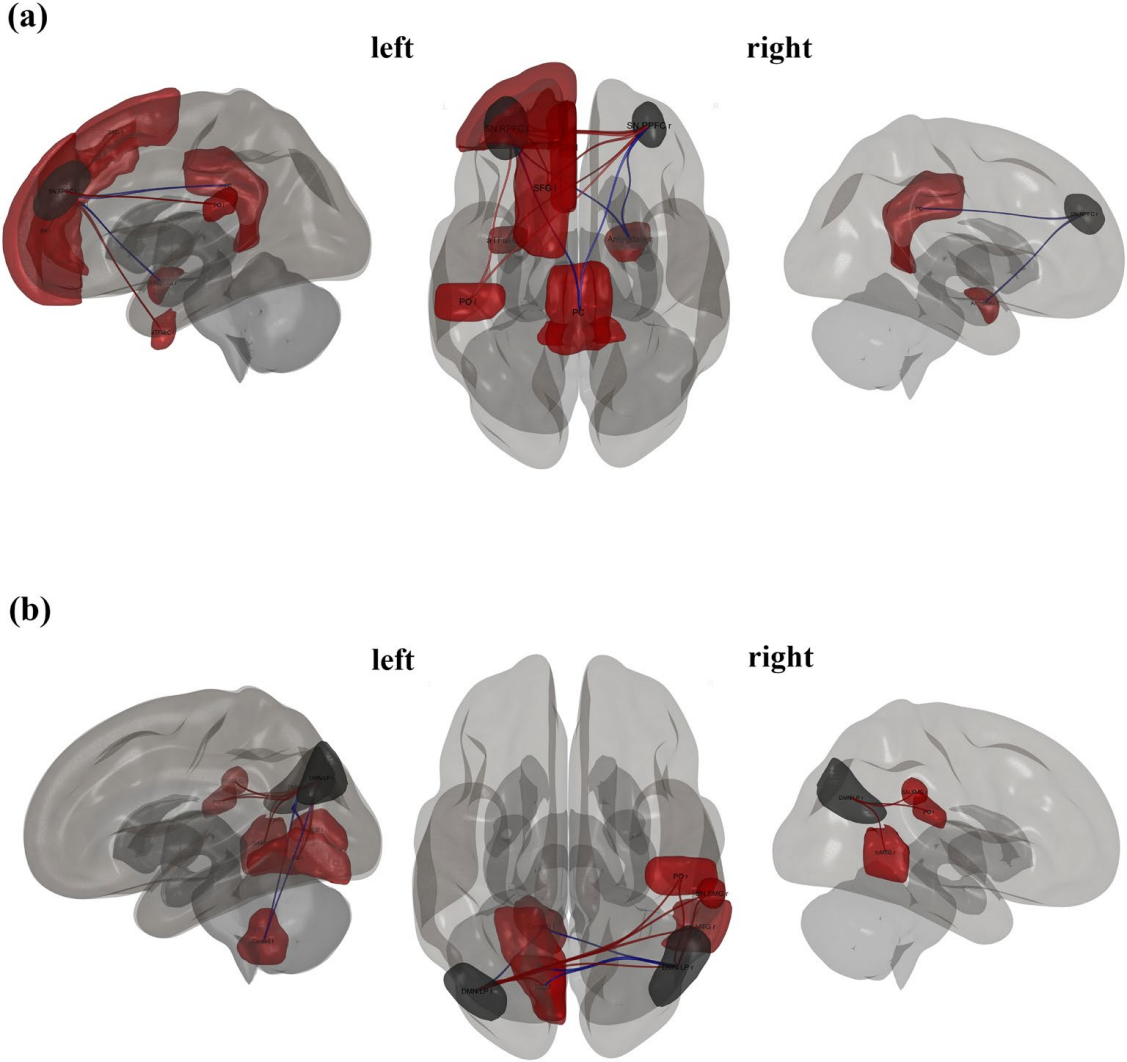
3D brain plots, specifically the seed ROI from SN.RPFC (a) and DMN.LP (b), which is known to correlate with other regions (*p*_unc_ < 0.05). The positive correlations are represented in red lines, and the negative correlations in blue lines, indicating the strength and direction of these associations, providing a visual representation of the brain's activity in relation to antisocial traits.

The seed regions and targets included ROIs that show significant connections for antisocial scores at an uncorrected *p*‐value of 0.05; see Table 4. The MPFC of the DMN showed significant positive associations with the Inferior Temporal Gyrus, temporooccipital right (*p*‐unc = 0.01) and significant negative associations with the Intracalcarine Cortex, right (*p*_unc_ = 0.03), as visualized in Figure 3b. Similarly, the ACC in the SN demonstrated significant positive associations with the LN Superior Temporal Gyrus, posterior right (*p*‐unc = 0.01) and the SN Rostral Prefrontal Cortex, right (*p*_unc_ = 0.02), among others, with *p*_unc_ values ranging from 0.02 to 0.04 for additional targets, as visualized in Figure 4b. Conversely, the left LPFC in the FPN revealed significant negative associations with the Cerebellum 10, Right (*p*_unc_ = 0.01) and positive associations with the Lateral Occipital Cortex, inferior left (*p*_unc_ = 0.02), while also encompassing targets with *p*_unc_ values of 0.04, as visualized in Figure 6b. The left RPFC in the SN exhibited significant negative associations with the Cingulate Gyrus, posterior (*p*_unc_ < 0.01) and positive associations with the right Amygdala (*p*_unc_ = 0.02), while also including targets with *p*_unc_ values of 0.04. On the other hand, the right RPFC in the SN showed significant positive associations with the anterior left Temporal Fusiform (*p*_unc_ < 0.01) and several other regions with *p*_unc_ values ranging from 0.02 to 0.04, as visualized in Figure 7a. Lastly, the left LP in the DMN showed significant negative associations with the left Cerebellum 9 (*p*_unc_ < 0.01) and a positive association with the SN.RPFC (*p*_unc_ = 0.04). In opposition, the right LP in the DMN exhibited significant positive associations with the right SN.SMG (*p*_unc_ = 0.01), the right Parietal Operculum (*p*_unc_ = 0.01), and the left Lingual Gyrus (*p*_unc_ = 0.01). Additionally, regions such as the LN.pSTG and toMTG r showed *p*_unc_ values of 0.03, while the FPN.LPFC and the pSTG r (*p*_unc_ = 0.04) were also associated (*p*_unc_ = 0.04), as visualized in Figure 7b.

**TABLE 3:** The seed regions and targets included ROIs that were significantly associated with narcissistic scores at an uncorrected *p*‐value of 0.05.

Seed	Targets	*b*	*t* (181)	*p*_unc_
The MPFC of the DMN	Temporal Pole, left (TP l)	0.01	3.14	< 0.01
	Frontal Orbital Cortext, right (FOrb r)	0.01	3.11	< 0.01
	Inferior Temporal Gyrus, posterior left (pITG l)	0.01	2.80	0.01
	SMN Superior	−0.01	−2.66	0.01
	Supracalcarine Cortext, right (SCC r)	−0.01	−2.63	0.01
	Precuneous Cortex (Precuneous)	−0.01	−2.63	0.01
	FPN lateral prefrontal cortex, right (FPN.LPFC r)	0.01	2.44	0.02
	Supramarginal Gyrus, posterior left (pSMG l)	0.01	2.42	0.02
	Middle Frontal Gyrus, right (MidFG r)	0.01	2.37	0.02
	Temporal Pole, right (TP r)	0.01	2.37	0.02
	DMN posterior cingulate cortex (DMN.PCC)	−0.01	−2.33	0.02
	FPN lateral prefrontal cortex left (FPN.LPFC l)	0.01	2.30	0.02
	SN. Anterior Insula, left (SN.Ainsula l)	0.01	2.29	0.02
	Postcentral Gyrus, right (PostCG r)	−0.01	−2.25	0.03
	LN Superior Temporal Gyrus, posterior (pSTG l)	0.01	2.25	0.03
	Inferior Temporal, posterior right (pITG r)	0.01	2.20	0.03
	Intracalcarine Cortex, right (ICC r)	−0.01	−2.20	0.03
	Middle Temporal Gyrus, anterior right (aMTG r)	0.01	2.17	0.03
	Middle Frontal Gyrus, left (MidFG l)	0.01	2.16	0.03
	Frontal Orbital Cortex, left (FOrb l)	0.01	2.15	0.03
	Supramarginal Gyrus, anterior right (aSMG r)	0.01	2.09	0.04
	Frontal Pole left (FP l)	0.01	2.05	0.04
The ACC in the SN	Paracingulate Gyrus, right (PaCiG r)	0.01	2.99	< 0.01
	Middle Temporal Gyrus, temporooccipital right (toMTG r)	0.01	2.66	0.01
	Superior Temporal Gyrus, posterior left (pSTG l)	0.01	2.43	0.02
	Frontal Pole right (FP r)	0.01	2.29	0.02
	Superior Frontal Gyrus left (SFG l)	0.01	2.01	0.04
The Ainsula in the SN	Frontal Pole, left (FP l)	0.01	2.46	0.01
	DMN Medial Prefrontal Cortex (DMN.MPFC)	0.01	2.29	0.02
The LPFC in the FPN	Juxtapositional Lobule Cortex, Left (SMA L)	−0.01	−3.08	< 0.01
	Superior Temporal Gyrus, anterior right (aSTG r)	0.01	2.89	< 0.01
	Temporal Fusiform Cortext, anterior left (aTFusC l)	0.01	2.64	0.01
	Cerebelum Crus2 Left (Cereb2 l)	−0.01	−2.56	0.01
	SMN Superior	−0.01	−2.38	0.02
	DMN Medial Prefrontal Cortex (DMN.MPFC)	0.01	2.30	0.02
	Temporal Pole Left (TP l)	0.01	2.25	0.03
	Vermis 7 (Ver7)	−0.01	−2.25	0.03
	Heschl's Gyrus right (HG r)	0.01	2.18	0.03
	Accumbens right (Accumbens)	0.01	2.17	0.03
	Superior Temporal Gyrus, right (pSTG r)	0.01	2.15	0.03
	Subcallosal Cortex (SubCalC)	0.01	2.09	0.04
	Cerebelum 7b Left (Cereb7 l)	−0.01	−2.08	0.04

Abbreviations: LN, Language Network; SMN, SensoriMotor Network.

**TABLE 4:** The seed regions and targets included ROIs that were significantly connected for antisocial scores at a 0.05 uncorrected *p*‐value.

Seed	Targets	*b*	*t* (181)	*p*_unc_
The MPFC of the DMN	Inferior Temporal Gyrus, temporooccipital right (toITG r)	0.01	2.58	0.01
	Intracalcarine Cortex, right (ICC r)	−0.01	−2.25	0.03
The ACC in the SN	LN Superior Temporal Gyrus, posterior right (LN.pSTG l)	0.01	2.84	0.01
	SN Rostral Prefrontal Cortex, right (SN.RPFC r)	0.01	2.41	0.02
	Thalamus right	0.01	2.37	0.02
	Inferior Temporal Gyrus, posterior right (pITG r)	0.01	2.34	0.02
	Middle Temporal Gyrus, temporooccipital right (toMTG r)	0.01	2.27	0.02
	Superior Temporal Gyrus, posterior left (pSTG l)	0.01	2.21	0.03
	Middle Temporal Gyrus, posterior left (pMTG l)	0.01	2.15	0.03
	Supramarginal Gyrus, posterior right (pSMG r)	0.01	2.15	0.03
The left RPFC in the SN	Cingulate Gyrus, posterior (PC)	−0.01	−3.73	0.00
	Amygdala, Right	0.01	2.42	0.02
	Vermis 9 (Ver9)	0.01	2.33	0.02
	Superior Frontal Gyrus, Left (SFG l)	−0.01	−2.31	0.02
	DMN posterior cingulate cortex (DMN.PCC)	−0.01	−2.28	0.02
	Amygdala, Left	< 0.01	2.09	0.04
	Ver10 (Vermis 10)	< 0.01	2.07	0.04
	Putamen, Right	< 0.01	2.07	0.04
	DMN Lateral Parietal, Left (DMN.LP l)	< 0.01	2.06	0.04
The right RPFC in the SN	Temporal Fusiform, anterior left (aTFusC l)	0.01	2.86	0.00
	Paracingulate Gyrus, Left (PaCiG l)	0.01	2.45	0.02
	Frontal Pole, Left (FP l)	0.01	2.43	0.02
	SN.ACC	0.01	2.41	0.02
	Vermis 45 (Ver45)	0.01	2.41	0.02
	Frontal Orbital C, Right (FOrb r)	0.01	2.37	0.02
	Parietal Operculum, Left (PO l)	−0.01	−2.30	0.02
	Thalamus, Left	0.01	2.19	0.03
	Amygdala, Right	< 0.01	2.17	0.03
	LN Inferior Frontal Gyrus, Right (LN.IFG r)	0.01	2.02	0.04
The left LPFC in the FPN	Cerebelum 10, Right (Cereb10 r)	−0.01	−2.81	0.01
	Caudate, Right	−0.01	−2.49	0.01
	Lateral Occipital Cortex, inferior left (iLOC l)	0.01	2.27	0.02
	Caudate, Left	−0.01	−2.13	0.03
	Cingulate Gyrus, posterior (PC)	0.01	2.11	0.04
The left LP in the DMN	Cereb9 l (Cerebelum 9 Left)	−0.01	−3.08	< 0.01
	SN.RPFC, Left	0.00	2.06	0.04
The right LP in the DMN	SN superior marginal gyrus, Right (SN.SMG r)	0.01	2.66	0.01
	Parietal Operculum, Right (PO r)	0.01	2.58	0.01
	Lingual Gyrus, Left (LG l)	−0.01	−2.50	0.01
	LN.pSTG, Right	0.01	2.24	0.03
	Middle Temporal Gyrus, temporooccipital right (toMTG r)	0.01	2.22	0.03
	Supramarginal Gyrus, anterior right (aSMG r)	0.01	2.21	0.03
	Intracalcarine Cortex, Left (ICC l)	0.00	−2.15	0.03
	FPN.LPFC, Right	0.01	2.06	0.04
	Superior Temporal Gyrus, Right (pSTG r)	0.00	2.05	0.04

Abbreviation: LN, Language Network.

## 4 Discussion

In our study, we employed a graph‐based network approach to identify macro‐network functionalities that could elucidate the complex connectivity patterns associated with narcissistic and antisocial traits. We hypothesized that global and regional measures of the DMN, SN, and FPN (i.e., the “triple network”) could predict both traits. This could provide evidence that these two personality types share not only symptoms but also similar brain characteristics.

As predicted, both traits were associated with reduced intra‐network connectivity within the SN, particularly in the ACC, and increased local efficiency (more effective processing) in the LPFC of the FPN, indicating similar altered patterns of emotional and cognitive control. Although we also confirmed a role for the DMN, this network displayed notable differences between the two traits. Narcissistic personality traits (NPT) were linked with increased DMN activity, especially in the medial prefrontal cortex (MPFC), while antisocial personality traits (APT) showed decreased DMN involvement. Higher betweenness centrality in the right RPFC of the SN and increased VN activity were observed only for APT.

These findings highlight both shared and distinct neural underpinnings, with the DMN's self‐reflective processes being more related to narcissism and the SN's emotional processes being more critical for antisocial traits. In the following sections, we will discuss these results in detail.

### 4.1 Shared Mechanisms

At a regional topological level, we found that the eccentricity measure of the ACC within the SN was predictive of both traits. Eccentricity refers to how much a node (in this case, the ACC) is connected to other nodes within the network (SN). The fact that eccentricity negatively predicts both traits indicates that the higher the personality traits, the lower the connectivity between the ACC and other regions of the SN. In other words, abnormal intra‐network connectivity in the SN is associated with both traits. This result aligns with previous evidence showing that altered GM in the ACC predicts narcissistic traits (Jornkokgoud et al. 2024, 2023). We also found that other hubs of the SN were affected in both traits, but with some differences. Specifically, the anterior insula (AI) of the SN was predictive of narcissistic traits, whereas the RPFC hub was more predictive of antisocial traits. Overall, the AI and the ACC are considered central nodes within the SN, known to respond to behaviorally salient events by integrating them with the emotional context (Jankowiak‐Siuda and Zajkowski 2013; Li et al. 2018; Sridharan et al. 2008). The AI and ACC are also active in response to experiences related to empathy, social rejection, high anxiety, or low self‐esteem, playing a major role in self‐ and other‐related emotional processing—especially relevant in the context of narcissistic traits (Cascio et al. 2014; Jankowiak‐Siuda and Zajkowski 2013; Jauk and Kanske 2021). Although the AI was not identified as a predictor of antisocial traits in our results, previous research has found reductions in GM volume and increased effective connectivity in the AI, as well as abnormal activation in individuals with antisocial behavior and a thinner‐than‐normal cortex in those with psychopathy (Aoki et al. 2014; Sitaram et al. 2014). In addition, the AI was highlighted as significantly involved in individuals with ASPD with psychopathy compared to those without psychopathy and non‐offenders, indicating distinct neural activation patterns between subtypes of ASPD (Gregory et al. 2015).

Regarding global topological metrics, we found that local efficiency in the lateral prefrontal cortex (LPFC) of the FPN was associated with both personalities. Higher efficiency in this region within the FPN correlated with higher narcissistic and antisocial traits. Specifically, individuals with higher trait levels exhibited increased local efficiency in this region, suggesting more efficient local processing and redundancy within the LPFC's neighborhood. The LPFC within the FPN is essential for executive control, integrating cognitive processes necessary for purposeful behavior. This region regulates attentional control, filters relevant information from distractions, and facilitates action selection to maintain focused attention and select behavioral targets (Hamilton et al. 2015; Tanji and Hoshi 2008). Additionally, the LPFC and MPFC are involved in higher‐order cognitive functions such as decision‐making and action planning, which are critical for the adaptive control of behavior (Hamilton et al. 2015; Nee and D'Esposito 2017; Tanji and Hoshi 2008). This result may indicate a greater capacity for planning and decision‐making, aiding in the pursuit of self‐serving goals, which is characteristic of both narcissistic and antisocial individuals.

We also found DMN involvement for both traits, although with opposite patterns (increased activity for narcissism and decreased activity for antisociality), and different subregions implicated (MPFC for narcissism and LPFC for antisociality). This suggests that both traits are encoded within the DMN, but with notable differences. The overexpression of the DMN in individuals with high narcissistic traits aligns with prior findings in other personality disorders (Grecucci et al. 2023, 2022; Langerbeck et al. 2023) and may underlie tendencies for overthinking and distorted self‐representations (Steiner et al. 2021). In contrast, reduced DMN activity in individuals with antisocial traits may reflect a diminished capacity for self‐reflection and more externally oriented thinking (Hamilton et al. 2015; Kılıçaslan et al. 2022).

Regarding the seed ROI analysis, the results showed that both narcissistic and antisocial traits involve overlapping regions within the SN and FPN, with significant connections, but not within the DMN. In the MPFC of the DMN, we found that connectivity associated with narcissistic traits significantly correlated with areas including the frontal, temporal, and cingulate cortices, while antisocial traits were primarily connected to regions in the temporal and occipital areas. These findings are consistent with our topological findings. Furthermore, the ACC within the SN was found to predict both traits, connecting with regions such as the right PaCiG, STG, right MTG, and right frontal lobe in relation to narcissism, while in antisocial traits, it connected with similar regions, including the temporal gyrus and the right thalamus. These results are consistent with previous findings showing the ACC's involvement in emotional regulation and reward processing (Dugré and Potvin 2022; Flannery et al. 2020). In contrast, the LPFC of the FPN was implicated in both traits, with correlations found between this region and the prefrontal cortex, motor cortex, and cerebellum. In narcissism, the LPFC was connected with the STG and anterior fusiform cortex (aTFusC), while in antisocial traits, it was connected with the cerebellum and cingulate gyrus. Previous evidence suggests that LPFC connectivity is crucial for cognitive control (Nee and D'Esposito 2017). Specifically, individuals with antisocial traits exhibit sub‐optimal FPN topology, likely linked to inefficient neural communication, which may contribute to difficulties in executive functioning (Lumaca et al. 2024). Our findings are consistent with prior studies demonstrating that GM and WM in regions like the MidFG, STG, and cerebellum contribute to the prediction of narcissistic traits (Jornkokgoud et al. 2024, 2023).

### 4.2 Distinct Mechanisms

The DMN (particularly the MPFC hub) was found to be more involved in narcissistic traits, but not in antisocial traits. This finding aligns with the differences in self‐reflective behavior between these two traits. Previous studies have suggested that narcissistic traits are reflected in the GM and WM networks contributing to the DMN (Jornkokgoud et al. 2024, 2023). Specifically, earlier research indicated that cortical volume in the MPFC within the DMN is negatively correlated with pathological narcissism (Mao et al. 2016). The MPFC is crucial for social cognition, which includes recognizing and understanding mental states, intentions, and emotions—both in oneself and in others (Le Petit et al. 2022). Abnormalities in cortical volume in this region are linked to deficits in these cognitive functions, which are essential for empathy and theory of mind (Mao et al. 2016; Massey et al. 2017).

In contrast, higher betweenness centrality in the right RPFC of the SN was solely likely to be associated with antisocial traits. Although RPFC is involved in higher‐level cognition, such as multitasking, goal maintenance, and integrating information from internal and external sources (Benoit et al. 2011; Friedman and Robbins 2022), its increased centrality in individuals with antisocial traits may not reflect better self‐regulation. Instead, it might support maladaptive cognitive processes, such as strategic planning for manipulative behaviors or goal‐directed aggression (Blair 2010, 2013). This is in line with evidence suggesting that individuals with antisocial tendencies are often characterized by poor impulse control and risk behaviors (Kernberg 1992). Conversely, narcissistic individuals, particularly those with grandiose features, are more often driven by self‐enhancement needs, attention seeking, and impulsive behaviors aimed at maintaining a positive self‐image, rather than cold, instrumental aggression (Miller et al. 2017).

Another notable difference between the two traits was the presence of the visual network in antisocial individuals, but not in narcissistic individuals. A recent study by Bakiaj et al. (2025) has suggested that individuals with high Dark Triad traits may display heightened visual abilities. Similarly, Espinoza et al. (2018) demonstrated that increased functional connectivity in the visual network, along with the SN and DMN, is associated with individuals displaying antisocial behavior, particularly forensic populations (Espinoza et al. 2018). However, earlier studies like Philippi et al. (2015) did not find associations between the visual and auditory networks and psychopathy in prison inmates (Philippi et al. 2015). As expected, the sensorimotor network, which was used as a control in this study, did not predict either narcissistic or antisocial traits.

Our seed ROI results further showed that both narcissistic and antisocial traits involve different regions within the DMN and SN. In individuals with narcissistic traits, the MPFC region of the DMN was associated with various targets, including the temporal pole, frontal orbital cortex, inferior temporal gyrus, and precuneus. These findings are consistent with earlier research showing that abnormalities in the GM volume of the MPFC are linked to reward and addiction, as well as socio‐emotional processes such as empathy and emotional regulation (Myznikov et al. 2024; Pastor and Medina 2021; Rolls et al. 2020). Although the MPFC is also involved in antisocial traits, its connectivity pattern differs. In antisocial individuals, it connects with regions such as the right inferior temporal gyrus and intracalcarine cortex, which are associated with visual and sensory processing. This may indicate altered responses to social cues. Additionally, the anterior insula and ACC within the SN are more related to narcissistic traits, with these regions showing connections to areas like the paracingulate gyrus and superior temporal gyrus. These results align with the topological metrics discussed earlier and are supported by recent studies suggesting reduced amygdala functioning as a central hub in the global information flow in psychopathic individuals (Tillem et al. 2019).

### 4.3 Translational Implications

Our findings suggest common and distinct neural correlates for narcissism and antisociality, offering important insights for clinical practice, particularly in diagnostics and treatment planning when dealing with clinical populations. For example, DMN hubs were negatively correlated with antisocial traits and positively correlated with narcissism, possibly explaining the differences in self‐reflection and sense of self between the two traits. This could lead to improved diagnostics for NPD and ASPD, which are often comorbid (Kraus and Reynolds 2001; Widiger 2011). The similarities in brain connectivity in the SN and FPN for both traits may be linked to the fact that individuals with these traits tend to exhibit dangerous behaviors and decreased awareness of risks, along with improved strategic planning and manipulation to achieve their goals. Moreover, the moderate relationship between narcissistic and antisocial subscales indicates overlapping characteristics in terms of the PSDI. These suggest that the dimensions and items of narcissistic and antisocial personality tests should be correlated and could be used to design more targeted biological interventions for individuals with NPD and ASPD, tailored to their specific neural profiles. For example, transcranial direct current stimulation over the prefrontal cortex has been shown to reduce intentions to commit aggressive acts and enhance moral judgment (Choy et al. 2018; Dambacher et al. 2015); its potential as a biological intervention targeting prefrontal dysfunctions.

### 4.4 Conclusions and Limitations

In the present study, we found a clear involvement of the DMN, SN, and FPN in narcissistic and antisocial traits, expanding previous evidence of their role in other personality disorders (Cao et al. 2022; Feng et al. 2018; Yang et al. 2015). The DMN's involvement in self‐referential and introspective processes, the SN's role in detecting and integrating salient stimuli, and the FPN's role in regulating self‐oriented control underscore the complex interplay of these networks in shaping narcissistic and antisocial personality traits.

Our research does not come without limitations. First, this study only focused on resting‐state functional MRI data; future research could explore the fusion of functional and structural MRI data, such as gray or white matter. Second, the PSSI was used in our study to evaluate narcissism, and it did not distinguish between the grandiose and vulnerable subtypes. Future research on this issue should go deeper and explore potential distinctions between the two forms of narcissism in terms of the triple brain network. Third, although we used the largest sample to date to our knowledge, future studies may benefit from increasing the sample size to obtain more robust results. Fourth, as gender differences were not the focus of this study, we excluded gender effects from our analyses to isolate the influence of personality traits on connectivity measures. Future studies could investigate potential gender differences in these personality traits. Lastly, although the seed‐to‐ROI connectivity analyses were guided by a priori selection of nodes identified through graph‐based metrics, the use of an uncorrected threshold (*p* < 0.05) increases the possibility of false positives. We therefore interpret these findings as exploratory and recommend that they be replicated using more conservative correction methods in future studies with larger samples.

In conclusion, our study confirms the involvement of the triple network in narcissistic and antisocial personalities, highlighting both similarities and differences; thereby advancing our understanding of this topic. These findings could inform the development of clinical interventions aimed at ameliorating these traits by targeting the functionality of the networks found to be altered.

## Author Contributions

**Khanitin Jornkokgoud:** conceptualization, formal analysis, investigation, methodology, project administration, visualization, validation, writing – original draft, writing – review and editing. **Richard Bakiaj:** data curation, writing – review and editing. **Peera Wongupparaj:** supervision, writing – review and editing. **Remo Job:** supervision, writing – review and editing. **Alessandro Grecucci:** conceptualization, methodology, supervision, writing – original draft, writing – review and editing.

## Conflicts of Interest

The authors declare no conflicts of interest.

## Data Availability

The data that support the findings of this study are openly available in OpenNeuro at https://doi.org/10.18112/OPENNEURO.DS000221.V2, reference number ds000221.
